# Overall reaction mechanism for a full atomic layer deposition cycle of W films on TiN surfaces: first-principles study†

**DOI:** 10.1039/c8ra07354f

**Published:** 2018-11-20

**Authors:** Hwanyeol Park, Sungwoo Lee, Ho Jun Kim, Daekwang Woo, Jong Myeong Lee, Euijoon Yoon, Gun-Do Lee

**Affiliations:** Department of Materials Science and Engineering, Seoul National University Seoul 08826 Korea eyoon@snu.ac.kr gdlee@snu.ac.kr; Department of Mechanical Engineering, Dong-A University Busan 49315 South Korea; Memory Thin Film Technology Team, Giheung Hwaseong Complex, Samsung Electronics 445-701 South Korea; Research Institute of Advanced Materials and Inter-University Semiconductor Research Center, Seoul National University Seoul 08826 South Korea

## Abstract

We investigated the overall ALD reaction mechanism for W deposition on TiN surfaces based on DFT calculation as well as the detailed dissociative reactions of WF_6_. Our calculated results suggest that the overall reactions of the WF_6_ on the B-covered TiN surfaces are energetically much more favorable than the one on the TiN surfaces, which means that the high reactivity of WF_6_ with the B-covered TiN surface is attributed to the presence of B-covered surface made by B_2_H_6_ molecules. As a result, an effect of the B_2_H_6_ flow serves as a catalyst to decompose WF_6_ molecules. Two additional reaction processes right after WF_6_ bond dissociation, such as W substitution and BF_3_ desorption, were also explored to clearly understand the detailed reactions that can occur by WF_6_ flow. At the first additional reaction process, W atoms can be substituted into B site and covered on the TiN surfaces due to the stronger bonding nature of W with the TiN surface than B atoms. At the second additional reaction process, remaining atoms, such as B and F, can be easily desorbed as by-product, that is, BF_3_ because BF_3_ desorption is an energetically favorable reaction with a low activation energy. Furthermore, we also investigated the effect of H_2_ post-treatment on W-covered TiN surface in order to remove residual F adatoms, which are known to cause severe problems that extremely degrade the characteristics of memory devices. It was found that both H_2_ dissociative reaction and HF desorption can occur sufficiently well under somewhat high temperature and H_2_ ambience, which is confirmed by our DFT results and previously reported experimental results. These results imply that the understanding of the role of gas molecules used for W deposition gives us insight into improving the W ALD process for future memory devices.

## Abstract 2

We investigated the overall ALD reaction mechanism for W deposition on TiN surfaces based on DFT calculation as well as the detailed dissociative reactions of WF_6_.

## Graphical abstract

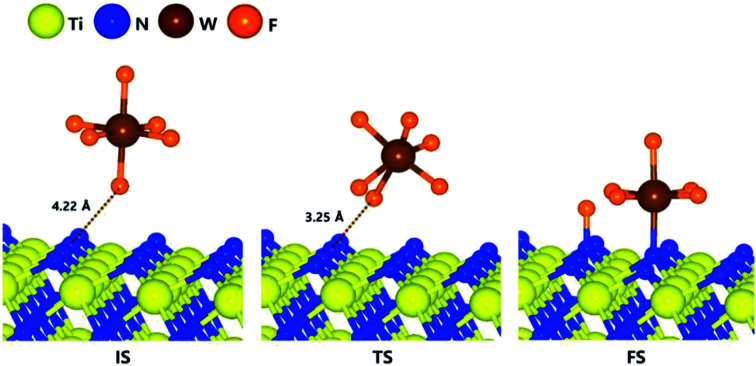

## 1. Introduction

Following Moore's law for decades, thin film deposition techniques have been intensively advanced to meet the demand for miniaturized and highly integrated devices in the electronics industry.^1^ Recently, conformal film deposition techniques, which allow precise thickness control at the atomic scale, are becoming very important.^2^ Nitride materials, such as titanium nitride and silicon nitride, have been deposited using conventional deposition methods such as low-pressure chemical vapor deposition (LPCVD)^3,4^ and plasma-enhanced chemical vapor deposition (PECVD).^5,6^ However, development of memory devices has required another deposition technique such as atomic layer deposition (ALD)^7–9^ to meet the demand for excellent step coverage and high conformality on extremely high aspect ratio structures. The ALD processes utilize well-controlled sequential surface reactions to obtain uniform and conformal films.^10,11^

As one of the most essential materials in fabrication of future memory devices, tungsten (W) has been used as a metal gate with lower resistivity than other candidate materials, which results in enhancement of device performance.^12,13^ In the fabrication of recent memory devices, tungsten films have been deposited using ALD by alternatively exposing W precursors such as tungsten hexafluoride (WF_6_) and reducing agents such as diborane (B_2_H_6_) in an ABAB… sequence. In the ALD processes for W deposition, B_2_H_6_ dosing process can play an important role in deposition of W films with low resistivity and in removal of residual fluorine (F) atoms on the surface.^14–16^

However, as the size of the memory device becomes smaller and smaller, it becomes difficult to deposit W films having excellent step coverage and conformality due to a severe problem that a seam or void is formed in the process of filling the W metal gate. This problem is a primary obstacle of the development for future memory devices.^17,18^ To treat this problem, theoretical comprehension of the ALD process for W deposition is required due to the experimentally limited observations on the sub-nanometer scale. Although a few experimental results on ALD W have been reported, there has been no theoretical report on the overall reaction mechanism for ALD W process.

In our previous study,^19^ we reported that these severe problems, such as seam or void, in filling the W metal gate for memory devices would be attributed to the difference of deposition rate of W film depending on the orientations of TiN surfaces by analyzing dissociation reaction of B_2_H_6_ on three different TiN surfaces, such as TiN (001), Ti-terminated TiN (111), and N-terminated TiN (111) using density functional theory (DFT) calculation method. Since this previous study gives only information for B_2_H_6_ dosing process, we want to report how important the understanding of the overall ALD reaction mechanism could be for improving W deposition process.

Here, we present first-principles study based on DFT calculation to explore overall ALD reaction mechanism for W deposition on the underlying TiN surfaces well as the detailed dissociative reactions of WF_6_. From our DFT calculated results, the structure of TiN surfaces can be changed depending on exposure to gas molecules, such as B_2_H_6_, WF_6_, and H_2_. As a result, the changed structure of the TiN surfaces can have a significant impact on the ALD W process because the underlying surfaces can have significant effects on the characteristics of the subsequent W nucleation layers.^20,21^ The TiN surfaces have been widely utilized as a glue/barrier layer for subsequent W nucleation.^22^ Three different planes of TiN surfaces, TiN (001), Ti-terminated TiN (111), and N-terminated TiN (111) can be generated because poly-crystalline TiN layers with (001) and (111) preferred orientations were mainly observed in deposition of TiN films.^23,24^ Our previous results^19^ imply that B-covered surface can be generated very well by B_2_H_6_ flow especially on N-terminated TiN (111) surface rather than other TiN surfaces due to even higher reactivity of B_2_H_6_ on the former than the latter.

In this study, both N-terminated TiN (111) and B-covered N-terminated TiN (111) surfaces were selected to compare surface reactivities of WF_6_. At the first step, the WF_6_ decomposition processes on both N-terminated TiN (111) and B-covered N-terminated TiN (111) surfaces were carefully analyzed in order to investigate the effect of the B-covered surface made by B_2_H_6_. Then, at the second step, two additional reaction processes right after WF_6_ bond dissociation, such as W substitution into B site and BF_3_ desorption, were investigated to understand the detailed reactions that can occur by WF_6_ flow. At the final step, we also studied the effect of H_2_ post-treatment on W-covered N-terminated TiN (111) surface in order to remove residual F adatoms, which are known to cause severe problems that extremely degrade characteristics of memory devices. It is expected that the understanding of the role of gas molecules used for W deposition gives us insight into improving the W ALD process for future memory devices.

## 2. Computational methods

In our theoretical results, all DFT calculations were performed using Vienna ab initio simulation package (VASP) program with the Perdew–Burke–Ernzerhof (PBE) functional in the generalized gradient approximation (GGA).^25,26^ We used PBE-D2 functional^27^ based on projector augmented wave (PAW) method^28^ with a correction to the conventional Kohn–Sham DFT energy to treat the van der Waals (vdW) interactions for all TiN surface calculations. TiN surfaces with B1–NaCl structure were used as the reactive surfaces with the WF_6_ precursor.

The optimized lattice parameter of TiN was *a*_0_ = 4.259 Å, which overestimate somewhat the experimental value (*a*_0_ = 4.24 Å)^29^ since generally PBE functionals tend to overestimate the lattice parameters. For the N-terminated TiN (111) surface, a 5-layer slab of (2 × 2) supercell was considered with vacuum gaps of 25.6 Å in the *z* direction were included to avoid interactions between adjacent slabs. For comparison, the B-covered N-terminated TiN surface was considered with a 5-layer slab of (2 × 2) supercell with vacuum gaps of 24.5 Å. Valence orbitals were described by a plane-wave basis set with the cutoff energy of 400 eV. Electronic energies were calculated with a self-consistent-field (SCF) tolerance of 10^−4^ eV on the total energy. Ultrasoft Vanderbilt-type pseudopotentials^30^ were used to describe the interactions between ions and electrons. A 3 × 3 × 3 Monkhorst *k*-point mesh for bulk TiN was chosen. The Brillouin zone for all TiN surfaces was sampled with a 3 × 3 × 1 Monkhorst–Pack *k*-point mesh. Geometry optimization was performed by minimizing the forces of all atoms to less than 0.02 eV Å^−1^ with the total energy of the system converged to within 10^−4^ eV during self-consistent iterations. In addition, we have calculated total energies for various configurations to determine the energy barrier for dissociative reactions of WF_6_ on both TiN surfaces in the first step, for both W substitution and BF_3_ desorption in the second step, and for both H_2_ dissociative reaction and HF desorption in the final step. This procedure for calculation of transition state is required to find not only the accurate final state but also transition state especially in complicated system, such as WF_6_ dissociation. After this procedure, we used nudged elastic band (NEB) method^31^ using the calculated final state to check accurate transition state. To optimize adsorption structures, we considered two orientations and three positions of WF_6_ on the N-terminated TiN surface. The details of all six cases are shown in the ESI (Fig. S4†). The optimized adsorption structures with the lowest energy in the ESI (Table S2†) were used in this paper. We also checked two orientations and thirteen positions of WF_6_ on the B-covered N-terminated TiN (111) surface. The details of all twenty-six cases are shown in the ESI (Fig. S5†). The optimized adsorption structures with the lowest in the ESI (Table S3†) were used in this paper.

## 3. Results and discussion

Charge density distribution for prediction of the surface reactivity of WF_6_ precursor was described in the ESI (Fig. S1–S3 and Table S1†)

### 3.1. WF_6_ dissociative chemisorption on N-terminated TiN (111)

**Fig. 1:**
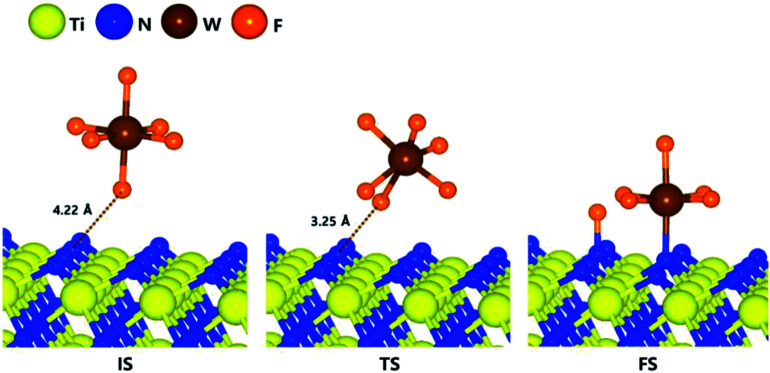
The optimized initial, transition, final structures for the first dissociative reaction step of WF_6_ on the N-terminated TiN (111) surface.

For ALD reaction to proceed, the WF_6_ precursor first undergo dissociative reaction on the TiN surfaces. The optimized structures of initial, transition, and final states for the first dissociative reaction step of WF_6_ molecule on the N-terminated TiN (111) surface are shown in Fig. 1. The calculated overall energy diagram of WF_6_ decomposition on the N-terminated.

In order to check the preference of F atom for the hollow site (on top of Ti atom) and the N site on the N-terminated TiN (111) surface, we have checked adsorption characteristics of one F atom on Ti site of the nitride surface. During the structural relaxation, we found that the F atom moved from the hollow site (on top of Ti atom) to N site on the surface. As seen in side-view of Fig. S4,† Ti atoms underneath N atoms on the surface would be hard to interact with F atom due to the Ti atoms.

**Fig. 2:**
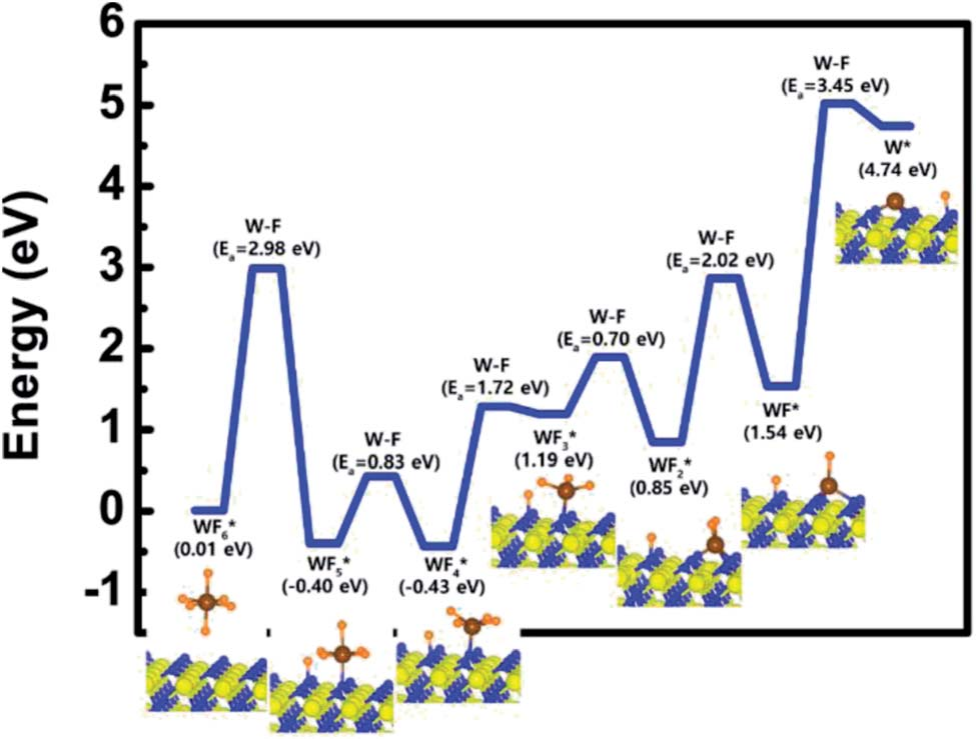
Calculated energy diagram of WF_6_ decomposition on the N-terminated TiN (111) surface.

TiN (111) surface is shown in Fig. 2. The initial state (IS) in Fig. 1 shows the optimized structure with the lowest adsorption energy of WF_6_ on the surface, which is 0.01 eV. The final state (FS) presents that the dissociated F atom from the WF_6_ molecule reacts with the N atom of the surface, and remaining WF_5_ reacts with another N atom of the surface. The reaction energy can be calculated as the energy difference between the initial state and the final state. As shown in Fig. 2, the calculated reaction energy of WF_6_ for the first reaction step on the N-terminated TiN (111) surface is −0.39 eV. The first reaction step is kinetically difficult due to a high activation energy of 2.98 eV.

To complete the overall reaction energetics of WF_6_, the calculated energy diagram of WF_6_ decomposition on the N-terminated TiN (111) surface is depicted in Fig. 2. The detailed structures of WF_6_ during the overall reactions on the N-terminated TiN (111) surface for transition state calculations can be found in the ESI (Fig. S6 and Table S4†). During the dissociative reactions of the WF_6_ molecule on the surface, this calculation shows that the overall reaction process is endothermic, with a calculated overall reaction energy of 4.73 eV. These results indicate that the reaction is thermodynamically unfavorable due to the uphill reactions. Furthermore, WF_6_ dissociative chemisorption on the N-terminated TiN (111) is kinetically difficult due to high activation energies that range from a minimum of 0.70 eV to a maximum of 3.45 eV. The low reactivity of WF_6_ with the N-terminated TiN (111) surface might be attributed to weak N–F bonding nature, which makes it difficult for W–F bond breaking of WF_6_ to occur. The weak N–F bonding nature may be originated from electron-withdrawing nature of both atoms due to high electronegativity of them (N = 3.04, F = 3.98), which causes N–F bonding nature to weaken, while strong bonding nature of B–N (B–N bond length = 1.48 Å) in Table S1† may be attributed to the electron-donation nature of B atom and the electron-withdrawing nature of N atom, which causes B–N bond to strengthen.^32^

### 3.2. WF_6_ dissociative chemisorption on B-covered N-terminated TiN (111)

**Fig. 3:**
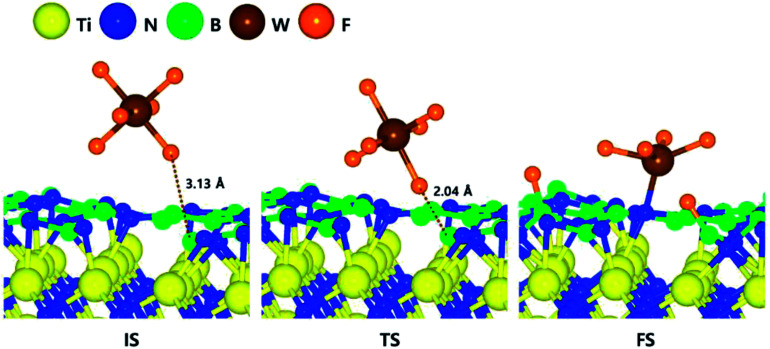
The optimized initial, transition, final structures for the first dissociative reaction step of WF_6_ on the B-covered N-terminated TiN (111) surface.

After B_2_H_6_ flow on the N-terminated TiN (111) surface, B-covered N-terminated TiN (111) surface can be made due to energetically favorable reaction of B_2_H_6_ bond dissociation on the N-terminated TiN surface, which was confirmed by our previous report.^19^ In order to estimate the difference between the N-terminated TiN (111) and B-covered N-terminated TiN (111) surfaces, the decomposition mechanism of WF_6_ was also studied on the B-covered N-terminated TiN (111) surface. The optimized structures of initial, transition, and final states for the first dissociative reaction step of WF_6_ molecule are shown in Fig. 3. It was found that dissociated F atoms were adsorbed on the B atoms, and remaining WF_4_ species was adsorbed on the N atom.

### 3.3. WF_6_ dissociative chemisorption on B-covered N-terminated TiN (111)

After B_2_H_6_ flow on the N-terminated TiN (111) surface, B-covered N-terminated TiN (111) surface can be made due to energetically favorable reaction of B_2_H_6_ bond dissociation on the N-terminated TiN surface, which was confirmed by our previous report.^19^ In order to estimate the difference between the N-terminated TiN (111) and B-covered N-terminated TiN (111) surfaces, the decomposition mechanism of WF_6_ was also studied on the B-covered N-terminated TiN (111) surface. The optimized structures of initial, transition, and final states for the first dissociative reaction step of WF_6_ molecule are shown in Fig. 3. It was found that dissociated F atoms were adsorbed on the B atoms, and remaining WF_4_ species was adsorbed on the N atom. The lowest adsorption energy of WF_6_ on the B-covered N-terminated TiN (111) surface is −0.10 eV, showing that the adsorption is energetically favorable. The reaction energy from IS to FS during the first dissociative step of WF_6_ is −0.28 eV, indicating that the reaction is exothermic with low activation energy of 0.19 eV.

**Fig. 4:**
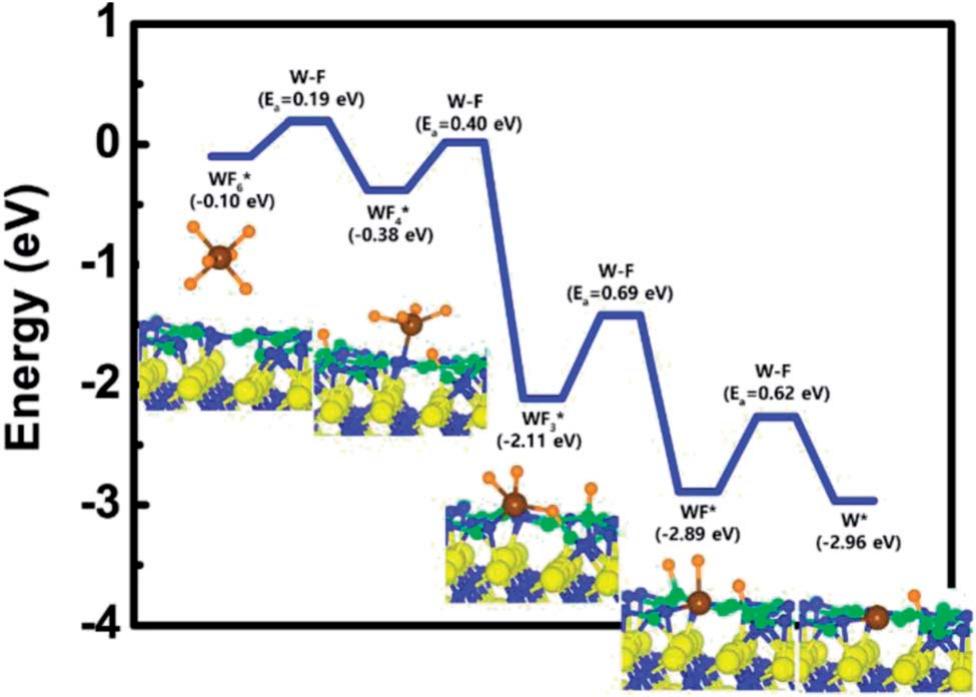
Calculated energy diagram of WF_6_ decomposition on the B-covered N-terminated TiN (111) surface.

To complete the overall reaction energetics of WF_6_, the calculated energy diagram of WF_6_ decomposition on the B-covered N-terminated TiN (111) surface is shown in Fig. 4. The detailed structures of WF_6_ during the overall reaction pathway on the surface for transition state calculations can be found in the ESI (Fig. S7 and Table S5†). During the reaction of the WF_6_ molecule, this calculation shows that the overall reaction process is exothermic, with a calculated overall reaction energy of −2.86 eV. This result indicates that WF_6_ dissociative chemisorption on B-covered N-terminated TiN (111) is energetically favorable due to the downhill reactions and low activation energies that range from a minimum of 0.19 eV to a maximum of 0.69 eV. WF_3_ species has the highest activation energy of W–F bond dissociation among WF_*x*_ species, which implies that the W–F bond dissociation of WF_3_ is the rate-determining step along the overall reaction.

Our calculated results suggest that the high reactivity of WF_6_ with the surface is attributed to the presence of B-covered surface made by B_2_H_6_ with compared to bare surface as discussed above Section 3.2, proving that dissociative reaction of WF_6_ is energetically unfavorable on the N-terminated TiN (111) surface. Aforementioned in Section 3.1, B-covered surface can have even larger reactivity with WF_6_ molecule than the case of bare surface since the B atoms that lose electron can easily react with F atoms that acquire electrons, which induces W–F bond breaking of WF_6_ to occur easily. This analysis was confirmed by higher binding energy of F adatom with the B-covered N-terminated TiN (111) than N-terminated TiN (111), the former is 6.3 eV and the latter is 3.6 eV in our calculations. As a result, an effect of the B_2_H_6_ flow on the surface is to make the TiN surface be reactive for WF_6_ bond dissociation, meaning that the B_2_H_6_ serves as a catalyst to decompose WF_6_.

### 3.4. Two additional reaction processes right after WF_6_ bond dissociation; W substitution and BF_3_ desorption

**Fig. 5:**
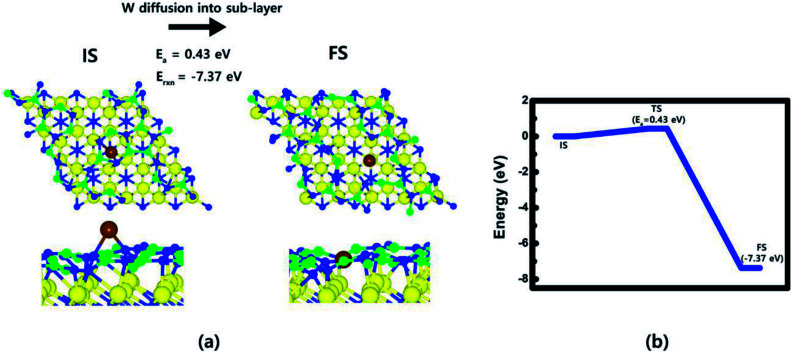
W adatom diffusion from atop (IS) to sub-layer (FS): (a) top and side views of W adatom on B-covered N-terminated TiN (111) surfaces. (b) Energy profile.

Right after WF_6_ dissociative reaction on B-covered N-terminated TiN (111), two additional reaction processes can occur; W substitution and BF_3_ desorption. For the first additional reaction process, the diffusion barrier of W adatom from atop (IS) to sub-layer (FS) was calculated to see if the W adatom can be substituted by B atoms in the sub-layer. As shown in Fig. 5(a), it was found that during W adatom diffusion from IS to FS, B–N bonded atoms just below the W adatom were broken and pushed out to bond with other B, N atoms in the vicinity. This phenomenon is attributed to strong binding energy of W adatom with the TiN surface than other B and N atoms (W: 10.9 eV, B: 5.9 eV, N: 9.4 eV, F: 4.4 eV) in our calculations. This gives new information why the TiN surface is greatly suitable for adhesion layer of W thin films. Fig. 5(b) shows W adatom diffusion from atop (IS) to sub-layer (FS) with a low activation energy of 0.43 eV and large reaction energy of −7.37 eV, which means that the diffusion is energetically favorable. This indicates that W atoms can be covered on the TiN surfaces due to the strong bonding nature of W with the TiN surface rather than B atoms covered on the surface. As a result, W-covered N-terminated TiN (111) surface can be made by an additional process of W substitution. In addition, remaining atoms, such as B and F, can be adsorbed on the W-covered N-terminated TiN (111) surface due to lower binding energy of those atoms with the surface than the one of W atom.

**Fig. 6:**
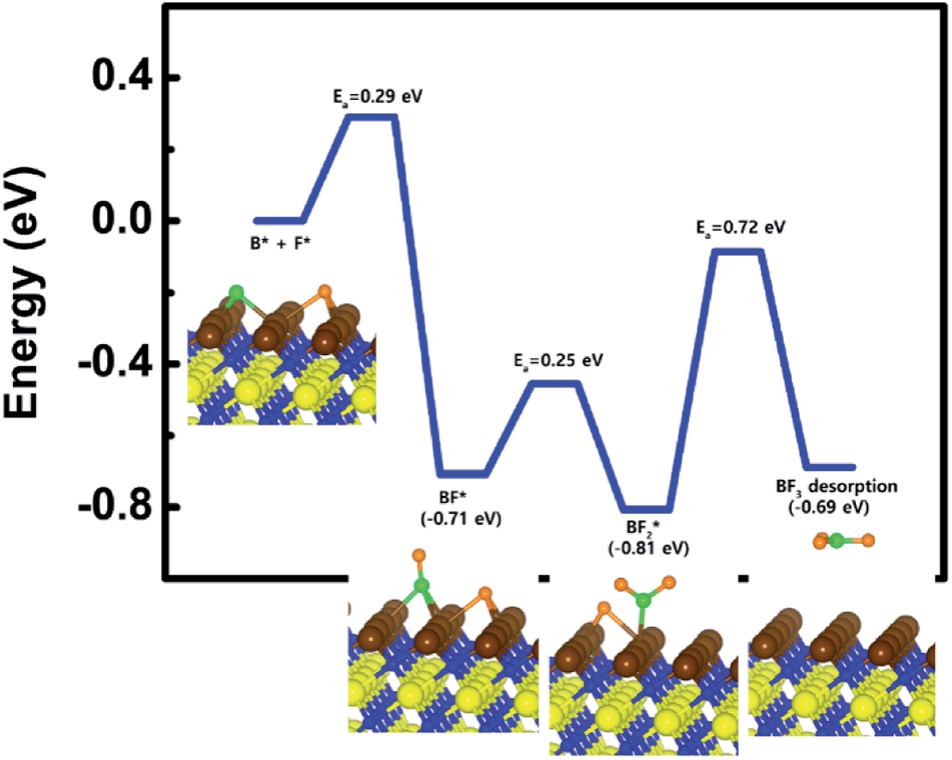
Calculated energy diagram of BF_3_ desorption on the W-covered N-terminated TiN (111) surface.

For the second additional reaction process, energy diagram of BF_3_ desorption was calculated to see if remaining B and F atoms can be desorbed as BF_3_ molecule. The calculated energy diagram of BF_3_ desorption on the W-covered N-terminated TiN (111) surface is shown in Fig. 6. The detailed structures during the overall reaction pathway on the surface for transition state calculations can be found in the ESI (Fig. S8 and Table S6†). During the reaction for BF_3_ desorption, this calculation shows that the overall reaction process is exothermic, with a calculated overall reaction energy of −0.69 eV. This result indicates that BF_3_ desorption on W-covered N-terminated TiN (111) is energetically favorable due to the downhill reactions and low activation energies that range from a minimum of 0.25 eV to a maximum of 0.72 eV. As a result, right after W substitution, remaining B and F atoms can be easily desorbed as by-product, that is, BF_3_ molecule on the W-covered N-terminated TiN (111) surface.

### 3.5. Effect of H_2_ post-treatment on W-covered N-terminated TiN (111)

Even though F adatoms can be removed by BF_3_ desorption, it may be difficult to completely remove F adatoms on the W-covered N-terminated TiN (111) surface because a large number of F adatoms can be generated right after WF_6_ dissociative reaction, which means that it is difficult to remove too many F adatoms by BF_3_ desorption. In fact, these residual F adatoms cause severe problems, such as, attack on under-layer materials and degradation of performance of memory devices, which is because F atoms with highly electron withdrawing nature have strong reactivity on other atoms with electron donation nature, such as Al, B, Si, *etc.*, leading to breaking the atomic bond of under-layer materials.^33,34^ In order to see if F adatoms can be removed by HF desorption, we investigated the effect of H_2_ post-treatment on W-covered N-terminated TiN (111) surface.

**Fig. 7:**
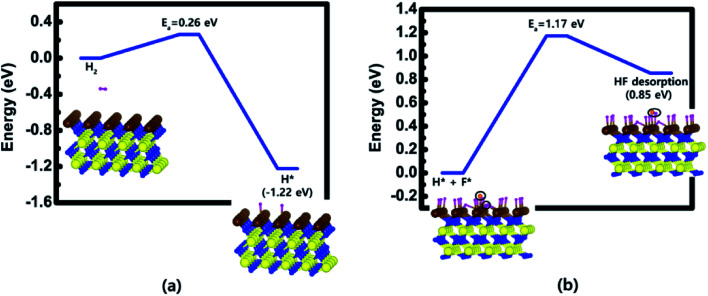
Calculated energy diagram of (a) H_2_ dissociation on the W-covered N-terminated TiN (111) surface and (b) HF desorption on the H-saturated W-covered N-terminated TiN (111) surface.

The calculated energy diagrams of H_2_ dissociation on the W-covered N-terminated TiN (111) and HF desorption on the H-saturated W-covered N-terminated TiN (111) surface are shown in Fig. 7(a) and (b), respectively. As for the first reaction step in Fig. 7(a), it shows that H_2_ dissociative reaction is energetically favorable, with a reaction energy of −1.22 eV and a low activation energy of 0.26 eV. This indicates that the injection of H_2_ molecule makes the W-covered N-terminated TiN (111) surface be the H-saturated surface.

As for the second reaction step in Fig. 7(b), it shows that HF desorption is energetically unfavorable, with a reaction energy of 0.85 eV and an activation energy of 1.17 eV. However, the forward reaction [H(s) + F(s) → HF(g)] can be increased under H_2_ ambience since large amount of H adatoms generated by H_2_ dissociation can increase the frequency of the forward reaction, which results in enhancing the reaction rate. In addition, the forward reaction may occur well under somewhat high temperature due to a bit high activation energy. In previous experimental study, Berç Kalanyan *et al.*^35^ reported that adding H_2_ with WF_6_ molecule promotes HF formation during the ALD process of W deposition. The combination of their results and our results provides how to effectively remove residual F atoms on the TiN surface.

### 3.6. Proposed overall ALD reaction mechanism for W deposition

**Fig. 8:**
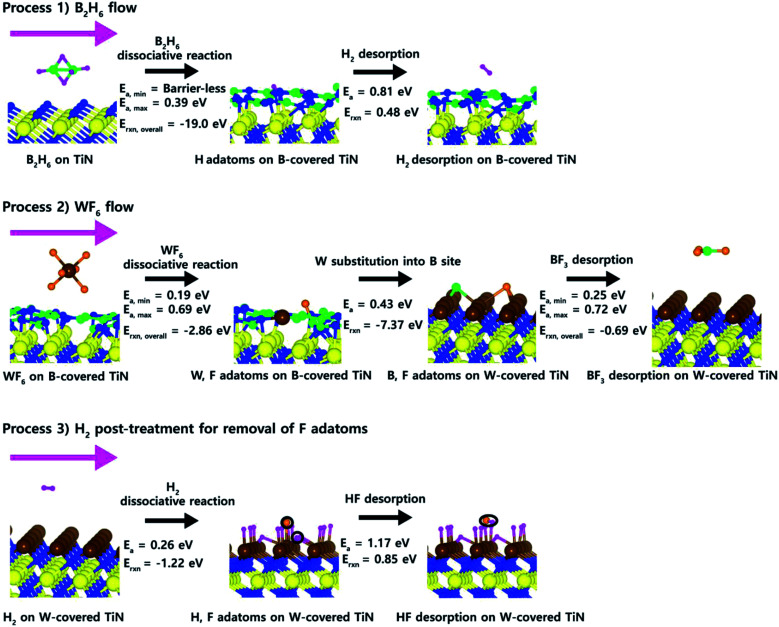
Proposed overall ALD reaction mechanism for W deposition.

In order to summarize overall ALD reaction mechanism for W deposition as above discussed in Section 3.1–3.5, we drew the overall ALD reaction scheme for clear understanding of W deposition. Fig. 8 shows proposed overall ALD reaction scheme for W deposition. At the process 1 for B_2_H_6_ flow, there are two reaction steps; B_2_H_6_ dissociative reaction and H_2_ desorption. It shows that B_2_H_6_ dissociative reaction is easy to occur on the N-terminated TiN (111) due to low activation energies (*E*_a,min_ = barrier-less, *E*_a,max_ = 0.39 eV) and overall reaction energy (*E*_rxn,overall_ = −19.0 eV), which was reported in our previous results.^19^ Therefore, B-covered N-terminated TiN (111) surface can be made by B_2_H_6_ flow. The remaining H adatoms on the surface can be desorbed as H_2_ molecule with activation energy (*E*_a_ = 0.81 eV) and reaction energy (*E*_rxn_ = 0.48 eV) under B_2_H_6_ ambience because large amount H adatoms generated by B_2_H_6_ dissociation can increase the frequency of the forward reaction [H(s) + H(s) → H_2_(g)] even though the reaction energy is positive value. At the process 2 for WF_6_ flow, there are three reaction steps; WF_6_ dissociative reaction, W substitution, and BF_3_ desorption. It shows that all three reaction steps are easy to occur due to low activation energies and overall reaction energies as above discussed in Section 3.3–3.5. At the process 3 for the effect of H_2_ post-treatment, there are two reaction steps; H_2_ dissociative reaction, HF desorption. It shows that both H_2_ dissociative reaction and HF desorption can occur enough well as above discussed in Section 3.5, therefore, a uniform W film could be deposited by removing residual F adatoms, which extremely degrade device performance. Our results indicate that the understanding of the role of gas molecules used for W deposition plays an important role in improving the properties of the subsequent W nucleation layers during the W ALD process.

## 4. Conclusions

In summary, we investigated overall ALD reaction mechanism for W deposition on the TiN surfaces based on DFT calculation as well as the detailed dissociative reactions of WF_6_. Our calculated results suggest that the overall reactions of the WF_6_ on the B-covered TiN surfaces are energetically much more favorable than the one on the TiN surfaces, which means that the high reactivity of WF_6_ with the B-covered TiN surface is attributed to the presence of B-covered surface made by B_2_H_6_ molecule. As a result, an effect of the B_2_H_6_ flow serves as a catalyst to decompose WF_6_ molecule. Two additional reaction processes right after WF_6_ bond dissociation, such as W substitution and BF_3_ desorption, were also explored to clearly understand the detailed reactions that can occur by WF_6_ flow. At the first additional reaction process, W atoms can be substituted into B site and covered on the TiN surfaces due to the strong bonding nature of W with the TiN surface than B atoms. At the second additional reaction process, remaining atoms, such as B and F, can be easily desorbed as by-product, that is, BF_3_ because BF_3_ desorption is energetically favorable reaction with low activation energy. Furthermore, we also investigated the effect of H_2_ post-treatment on W-covered TiN surface in order to remove residual F adatoms, which are known to cause severe problems that extremely degrade characteristics of memory devices. It was found that both H_2_ dissociative reaction and HF desorption can occur enough well under somewhat high temperature and H_2_ ambience, which is confirmed by the our DFT results and previously reported experimental results. These results imply that the understanding of the role of gas molecules used for W deposition gives us insight into improving the W ALD process for future memory devices.

## Conflicts of interest

There are no conflicts to declare.

## Supplementary Material

RA-008-C8RA07354F-s001
