# Supplementary material for: Overall reaction mechanism for a full atomic layer deposition cycle of W films on TiN surfaces: first-principles study
Source: RSC Adv. 2018 Nov 20;8(68):39039–46. doi: 10.1039/c8ra07354f (PMC9090620; doi:10.1039/c8ra07354f)
Supplement: RA-008-C8RA07354F-s001 [file RA-008-C8RA07354F-s001.pdf]

Electronic Supplementary Material (ESI) for RSC Advances.
This journal is © The Royal Society of Chemistry 2018

Electronic Supplementary Information

Overall reaction mechanism for a full atomic layer deposition cycle of W films on TiN surfaces: First-principles study

Hwanyeol Park,[1] Sungwoo Lee,[1] Ho Jun Kim,[2] Daekwang Woo,[3] Jong Myeong Lee,[3] Euijoon Yoon,*[,1,4] Gun-Do Lee*[,1,4]

[1]Department of Materials Science and Engineering, Seoul National University, Seoul 08826, Korea.

[2]Department of Mechanical Engineering, Dong-A University, Busan 49315, South Korea.

[3]Memory Thin Film Technology Team, Giheung Hwaseong Complex, Samsung Electronics, 445-701, South Korea.

[4]Research Institute of Advanced Materials and Inter-University Semiconductor Research Center, Seoul National University, Seoul 08826, South Korea.

Corresponding authors: eyoon@snu.ac.kr; gdlee@snu.ac.kr

Figure S1 shows 3D electron density distribution of the optimized structures of WF_6 precursor at 0.025 $Å^{-3}$ isosurface. It shows charge depletion around the W atom with electron loss of 5.34e and accumulation (pink area) in the vicinity of the F atom with electron gain of -0.89. The amount of charge transfer was calculated by Bader charge analysis. Figure S2 (a) shows 3D electron density distribution of the optimized structures of the N-terminated TiN (111) surface at 0.025 $Å^{-3}$ iso-surface. The charge depletion around the Ti atom with electron loss of 2.43e and accumulation (pink area) in the vicinity of the N atoms with electron gain of -1.55e can be seen. Figure S2 (b) exhibits 2D electron density map for top-layer of the same surface. Figure S2 (a) and (b) indicate that the N atoms with a high electron accumulation can be more accessible for electrophilic attack by WF_6 precursor due to the higher electron density and higher surface exposure than Ti atoms.

Although the Ti atoms are somewhat positively charged, WF_6 precursor would predominantly interact with the N atoms of the first top-layer due to the shorter distance than the Ti atoms of the second top-layer. Figure S3 (a) shows 3D electron density distribution of the optimized structures of the B-covered N-terminated TiN (111) surface at 0.025 $Å^{-3}$ iso-surface. It shows charge depletion around the Ti and B atoms with electron loss of 2.19e and 2.21e, respectively.

Charge accumulation (pink area) in the vicinity of the N atoms with electron gain of -2.99e can be seen. 2D electron density map of top-layer of the same surface is displayed in Figure S3 (b). Figure S3 (a) and (b) indicate that the B atoms with a high electron depletion can be more accessible for nucleophilic attack by the lone pair electron on the F atom of WF_6 precursor, forming strong B-F bonding to be anchored on the B-covered N-terminated TiN (111) surface. The N atoms with a high electron accumulation can be electrophilic attacked by the W atom of the WF_6 precursor. Even though the Ti atoms are positively charged, WF_6 precursor would predominantly interact with the N and B atoms of the first top-layer due to the shorter distance than the Ti atoms of the second top-layer. Table 1 summarizes the bond lengths and amount of charge transfer of the optimized structures of N-terminated TiN (111), B-covered N-terminated TiN (111), and WF_6 precursor. Our results show that Ti-N bond length is elongated from 1.95 Å at the N-terminated TiN (111) to 2.13 Å at the B-covered N-terminated TiN (111) surface due to stronger B-N bonding nature (B-N bond length = 1.48 Å) than Ti-N one, which means that the amount of charge transfer between B and N atoms can increase, while decreasing the one between Ti and N atoms. This indicates that the surface reactivity of WF_6 precursor can be more favorable on the B-covered N-terminated TiN (111) surface than the former because the F atoms that acquire electrons can easily react with B atoms that lose electron, while W atom can react with N atom. In order to confirm the favorable reactivity of WF_6 on the B-covered N-terminated TiN (111) surface, overall reaction energetics for WF_6 bond dissociations were deeply analyzed in the following section.

This information explains the detailed structures of adsorption, transition, and reaction state of WF_6 on both N-terminated TiN and B-covered N-terminated TiN surfaces. The adsorption energy (E_{ads}) was calculated using

$E_{ads} = E_{tot,ads} - (E_{surf} + E_{pre})$

where $E_{tot,ads}$, E_{surf}, and E_{pre} are the total energy of the system after adsorption, and the energy of the surface only and the energy of the precursor only, respectively. The activation energy (E_a) was calculated using

$E_a = E_{tot,tran} - E_{tot,b.tr}$

where $E_{tot,tran}$ and $E_{tot,ads}$ are the total energy of the transition state and the total energy before transition, respectively. The reaction energy (E_{rxn}) was calculated using

$E_{rxn} = E_{tot,a.tr} - E_{tot,b.tr}$

where $E_{tot,a.tr}$, and $E_{tot,b.tr}$ are the total energies of the system after transition and after transition, respectively.

We considered two orientations and three positions of WF_6 on the N-terminated (111) TiN surface as shown in Figures S1. As for the two orientations, we construct two extreme geometries. Geometry 1 is that only one fluorine atom of octahedral WF_6 is facing towards the surface, and Geometry 2 is that two fluorine atoms are facing towards the surface. Also, three different positions were considered on the surface.

We also checked two orientations and thirteen positions of WF_6 on the B-covered N-terminated (111) TiN surface as shown in Figures S2. The two orientations are same to the former case. Also, thirteen different positions were considered on the surface.

The adsorption energies of WF_6 calculated on both N-terminated TiN (111) and B-covered N-terminated TiN (111) surfaces for each orientation and position were summarized in Table S1-S2.

Figures S3-S4 show the optimized structures at specific reaction states, such as initial, transition, and final step, for WF_6 decomposition on both N-terminated TiN (111) and B-covered N-terminated TiN (111) surfaces. Both initial and final states were calculated at the position of remaining W, F, and WF_x species on the most stable site of those surfaces. Since diffusion barrier energies of F atom on both N-terminated (111) TiN and B-covered N-terminated (111) TiN are somewhat low, 0.31 and 0.46 eV, respectively, we assume that the adsorbed F atoms diffuse far away from the adsorbed WF_x species on the TiN surfaces under W ALD process. Therefore, we didn't put the diffused F atom in the next reaction steps because we assume that the F atom is diffused away. Table S3-S4 indicate the activation energies and reaction energies for the overall reactions of WF_6 decomposition on both surfaces. These tables provide information on whether the WF_6 reaction is energetically stable or unstable for each reaction step.

Figure S5 shows the optimized structures at specific reaction states, such as initial, transition, and final step, for BF_3 desorption on W-covered N-terminated TiN (111) surface. Since diffusion barrier energies of F atom on the W-covered N-terminated (111) TiN is somewhat low, 0.42 eV, we assume that the adsorbed F atoms diffuse into B atom around by strong interaction of B-F on the TiN surfaces under W ALD process. Therefore, we didn't put the diffused F atom in the next reaction steps because we assume that the F atom is diffused into B atom around. Table S5 indicate the activation energies and reaction energies for the overall reactions of BF_3 decomposition on the TiN surfaces.

Figure S6 shows the optimized structures at specific reaction states, such as initial, transition, and final step, for H_2 dissociative reaction and HF desorption on W-covered N-terminated TiN (111) surface. Table S6 indicate the activation energies and reaction energies for H_2 bond dissociation and HF desorption on the W-covered N-terminate TiN (111) surface.

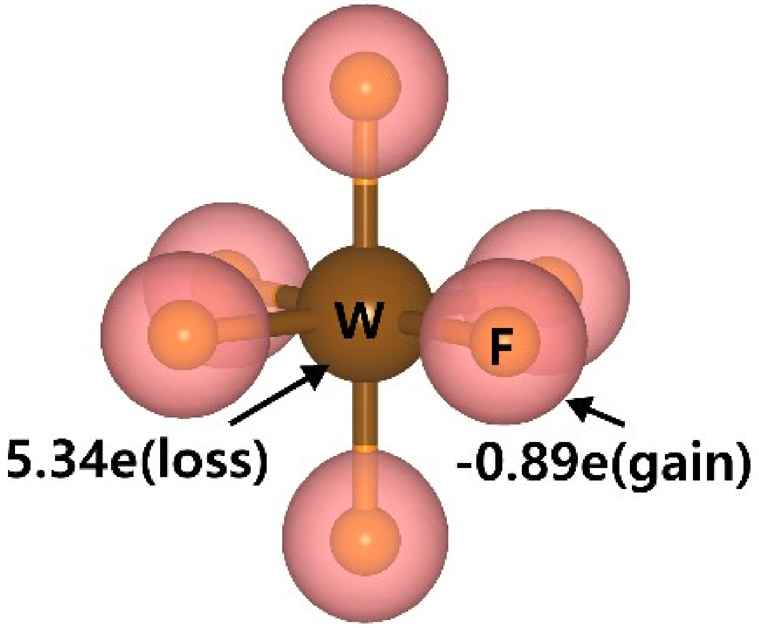

Figure S1. 3D electron density distribution of the optimized structure of the WF_6 precursor. Charge depletion around the W atom with electron loss of 5.34e and accumulation (pink area) in the vicinity of the F atom with electron gain of -0.98e can be seen.

Table S1. Bond lengths (Å) and amount of charge transfer (e) of the optimized structures for N-terminated TiN (111), B-covered N-terminated TiN (111), and WF_6 molecule.

	N-terminated TiN (111)	B-covered N-terminated TiN (111)	WF_6 molecule
Bond length (Å)	Ti-N: 1.95	Ti-N: 2.13<br>B-N: 1.48	W-F: 1.86
ΔQ (e)	Ti: 2.43<br>N: -1.55	Ti: 2.19<br>N: -2.99<br>B: 2.22	W: 5.34<br>F: -0.89

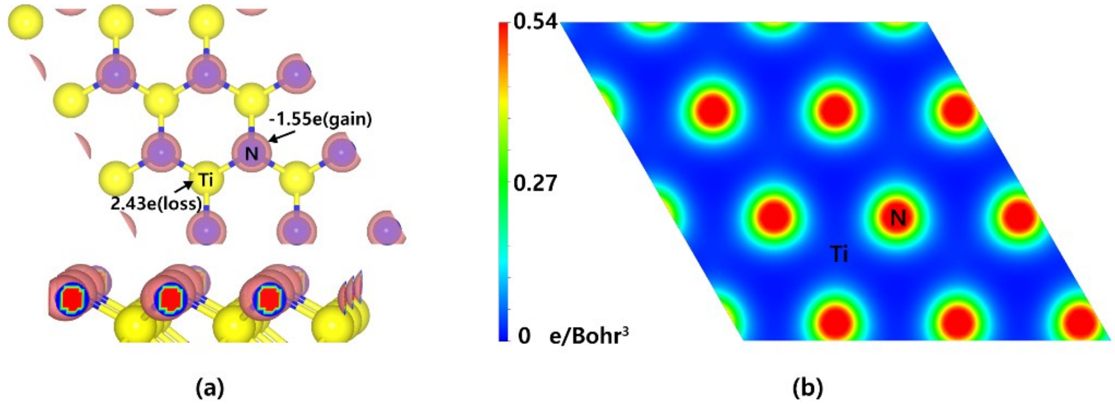

Figure S2. The optimized structure of the N-terminated TiN (111) surface: (a) 3D electron density of top and side views at 0.025 $Å^{-3}$ iso-surface. Charge depletion around the Ti atom with electron loss of 2.43e and accumulation (pink area) in the vicinity of the N atoms with electron gain of -1.55e can be seen. (b) 2D electron density map for top-layer of the same surface.

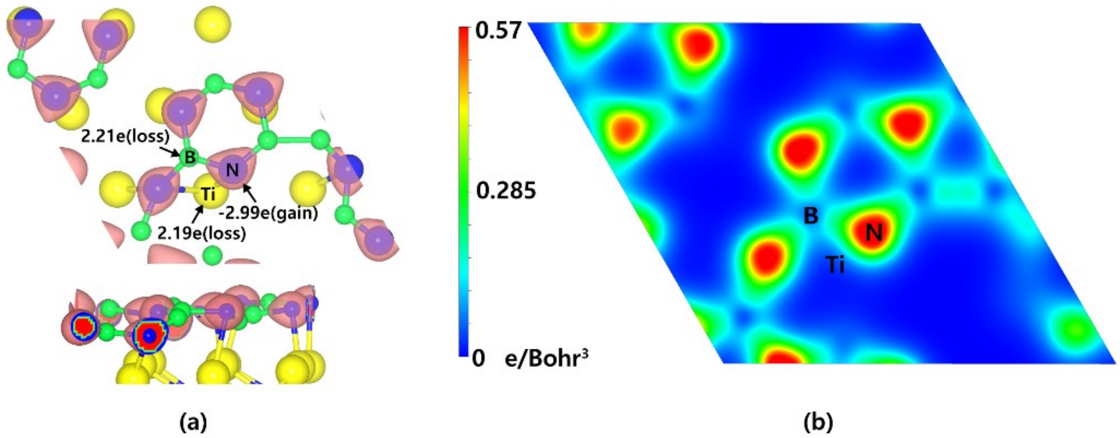

Figure S3. The optimized structure of the B-covered N-terminated TiN (111) surface: (a) 3D electron density of top and side views at 0.025 $Å^{-3}$ iso-surface. It shows charge depletion around the Ti atom and B atom with electron loss of 2.19e and 2.21e, respectively. Charge accumulation (pink area) in the vicinity of the N atoms with electron gain of -2.99e can be seen. (b) 2D electron density map for top-layer of the same surface.

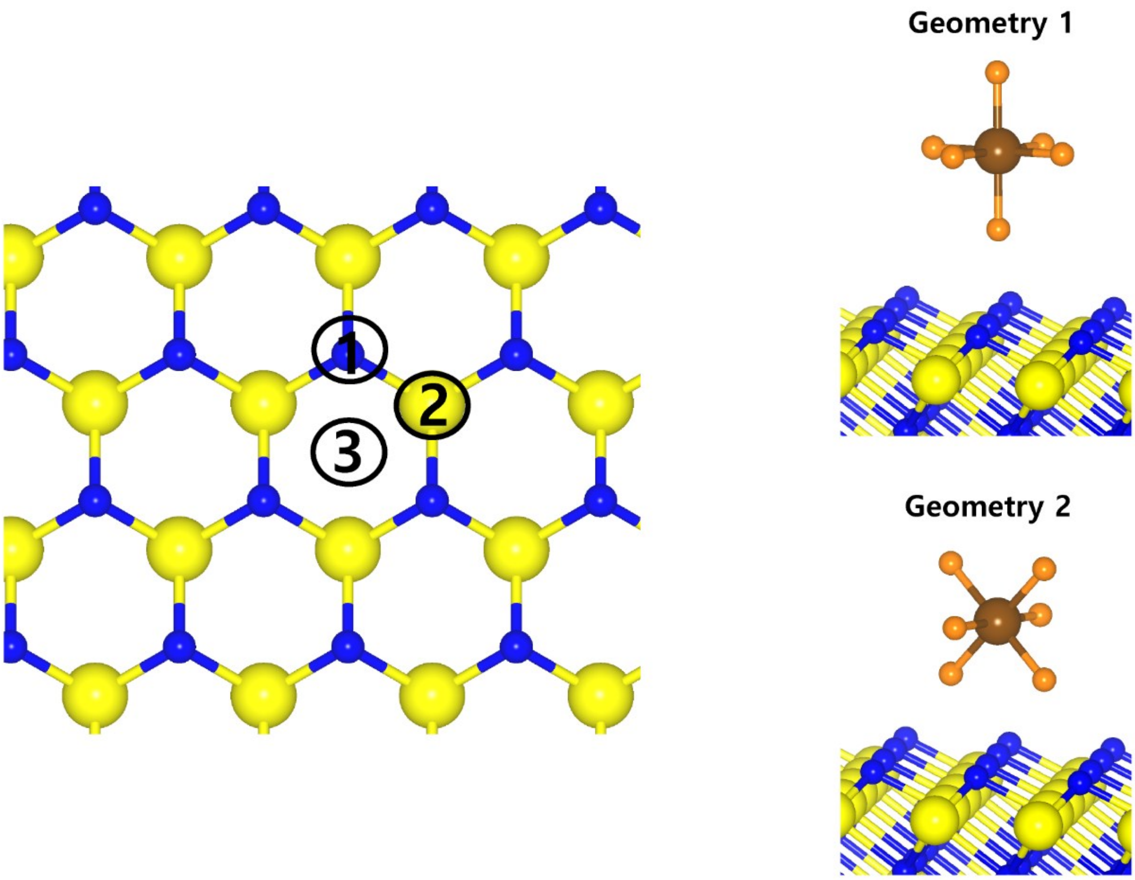

Figure S4. Two geometries and three positions of WF_6 on the N-terminated TiN (111) surface.

Table S2. The adsorption energies of WF_6 calculated on the N-terminated TiN (111) surface for each orientation and position.

Geometry	Position	E ads (eV)
1	1	0.05064
1	2	0.04548
**1**	**3**	**0.00926**
2	1	0.07967
2	2	0.03714
2	3	0.02117

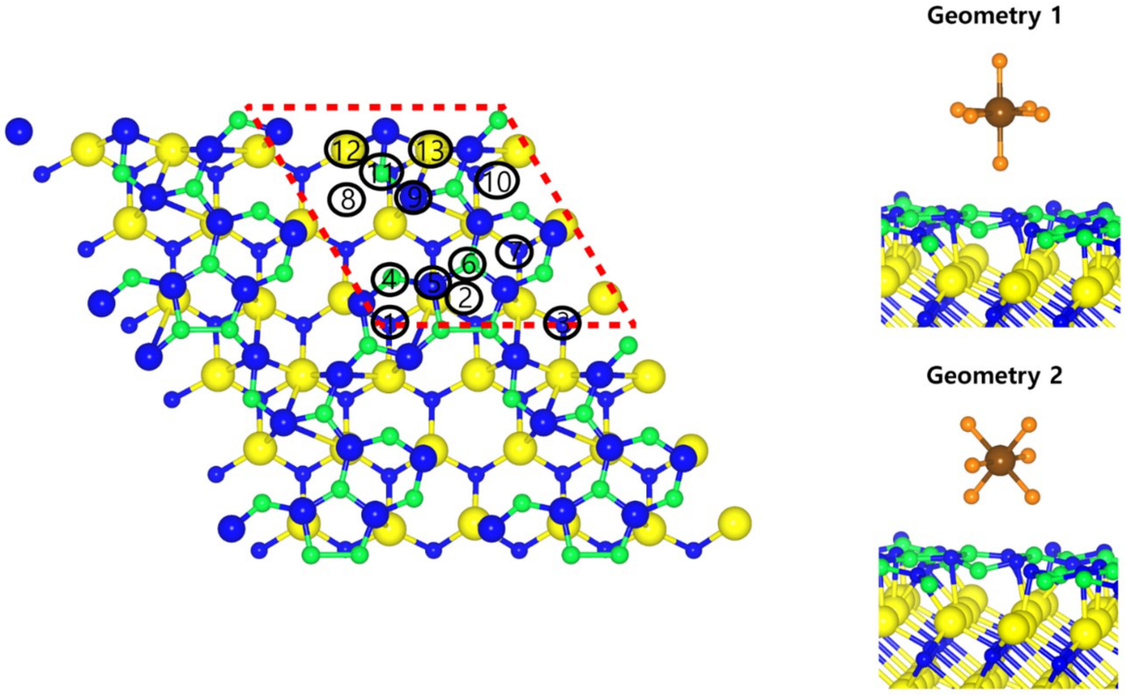

Figure S5. Two geometries and thirteen positions of WF_6 on the B-covered N-terminated TiN (111) surface.

Table S3. The adsorption energies of WF_6 calculated on the B-covered N-terminated TiN (111) surface for each orientation and position.

Geometry	Position	E ads (eV)
1	1	-0.04537
1	2	-0.06495
1	3	-0.05904
1	4	-0.0066
1	5	-0.05118
1	6	-0.06022
1	7	-0.05046
1	8	-0.07439
1	9	-0.05701
1	10	-0.02979
1	11	-0.08477
1	12	-0.08292
1	13	-0.02978
2	1	-0.08673
2	2	-0.08427
2	3	-0.07326
2	4	-0.07035
2	5	-0.07278
2	6	-0.05566
2	7	-0.06183
**2**	**8**	**-0.10041**
2	9	-0.04205
2	10	-0.07557
2	11	-0.08042
2	12	-0.05213
2	13	-0.06605

2nd reaction step : W-F bond dissociation

IS TS FS

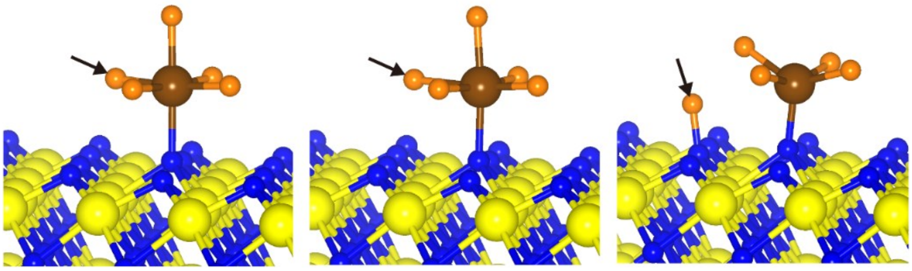

3rd reaction step : W-F bond dissociation

IS TS FS

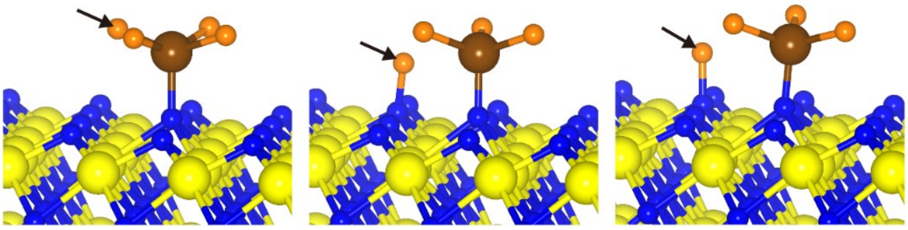

4th reaction step : W-F bond dissociation

IS TS FS

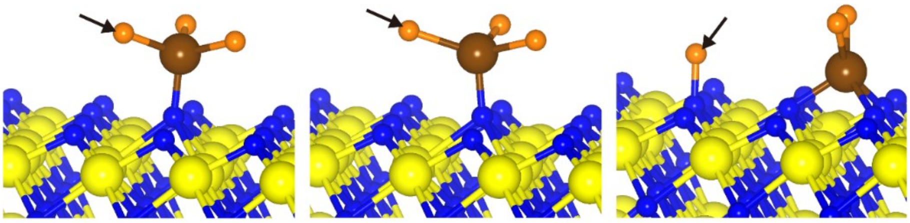

5th reaction step : W-F bond dissociation

IS TS FS

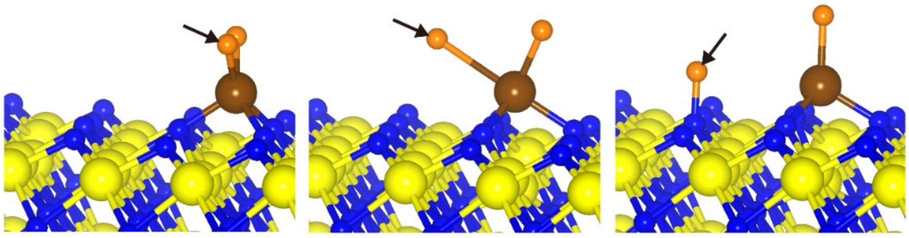

6th reaction step : W-F bond dissociation

IS TS FS

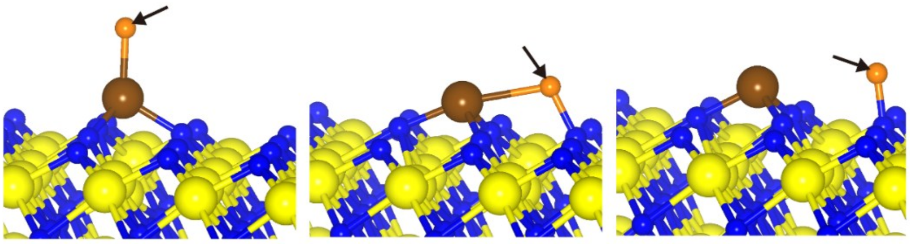

Figure S6. Initial (IS), transition (TS), and final (FS) states of intermediate reactions from 2nd reaction step to the 6th reaction step of WF_6 bond dissociation on the N-terminated TiN (111) surface.

Table S4. Activation energies (E_a, eV) and reaction energies (E_{rxn}, eV) of WF_6 bond dissociation on the N-terminated TiN (111) surface.

Reacton step	Bond dissociation	E_a (eV)	E_{rxn} (eV)
step 1	W-F	2.98	-0.39
step 2	W-F	0.83	-0.03
step 3	W-F	1.72	1.62
step 4	W-F	0.70	-0.34
step 5	W-F	2.02	0.69
step 6	W-F	3.45	3.20

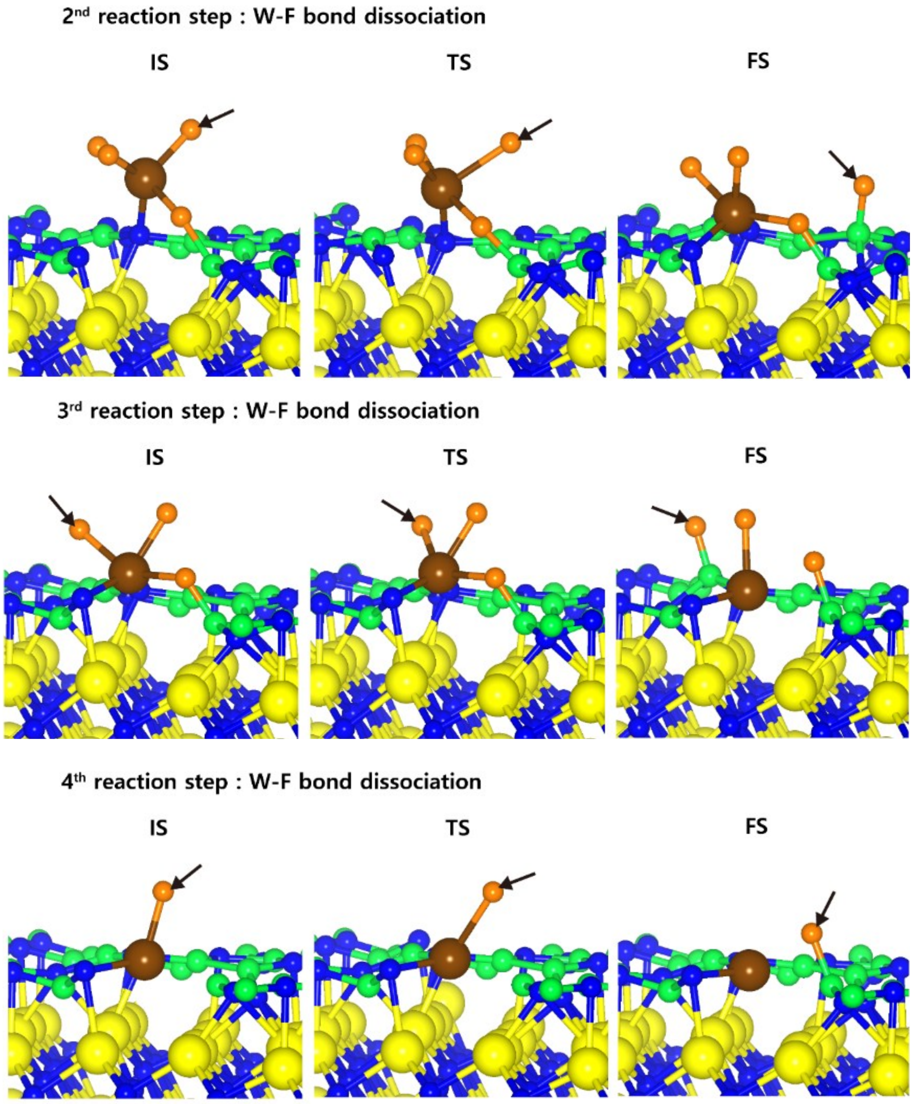

Figure S7. Initial (IS), transition (TS), and final (FS) states of intermediate reactions from 2nd reaction step to the 4th reaction step of WF_6 bond dissociation on the B-covered N-terminated TiN (111) surface.

Table S5. Activation energies (E_a, eV) and reaction energies (E_{rxn}, eV) of WF_6 bond dissociation on the B-covered N-terminated TiN (111) surface.

Reacton step	Bond dissociation	E_a (eV)	E_{rxn} (eV)
step 1	W-F	0.19	-0.28
step 2	W-F	0.40	-1.73
step 3	W-F	0.69	0.78
step 4	W-F	0.62	-0.07

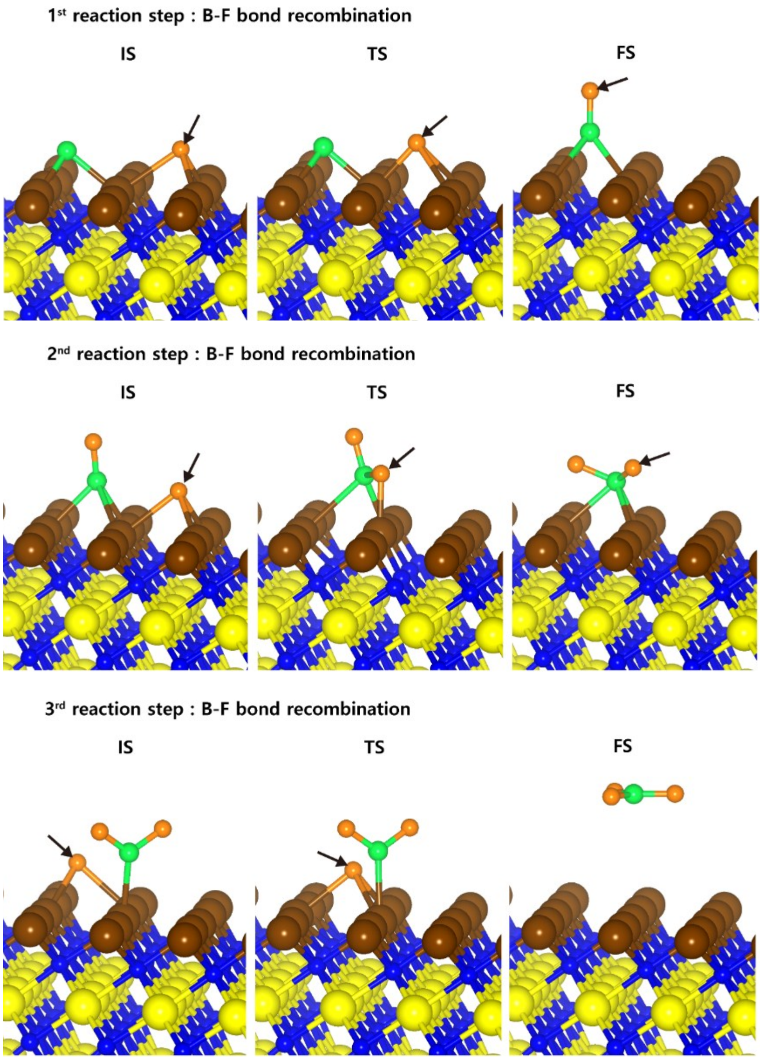

Figure S8. Initial (IS), transition (TS), and final (FS) states of intermediate reactions from 1st reaction step to the 3rd reaction step of BF_3 desorption on the W-covered N-terminated TiN (111) surface.

Table S6. Activation energies (E_a, eV) and reaction energies (E_{rxn}, eV) of B-F bond recombination on the W-covered N-terminated TiN (111) surface.

Reacton step	Bond recombination	E_a (eV)	E_{rxn} (eV)
step 1	B-F	0.29	-0.71
step 2	B-F	0.25	-0.10
step 3	B-F	0.72	0.12

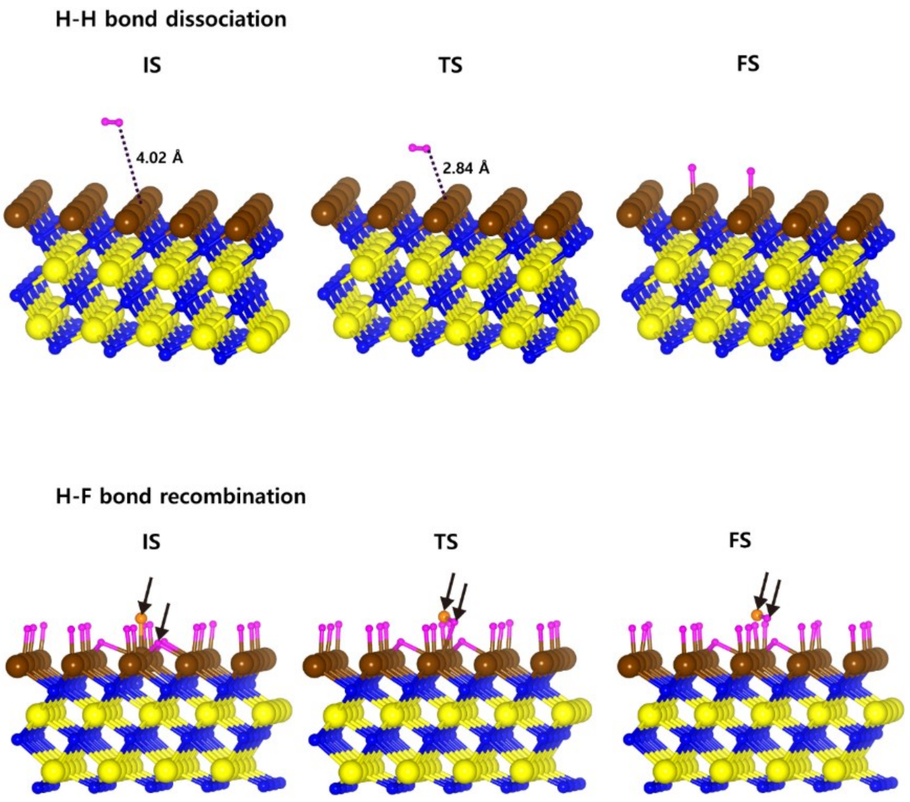

Figure S9. Initial (IS), transition (TS), and final (FS) states of H_2 bond dissociation and HF desorption on the W-covered N-terminated TiN (111) surface.

Table S7. Activation energies (E_a, eV) and reaction energies (E_{rxn}, eV) of H_2 bond dissociation and HF desorption on the W-covered N-terminated TiN (111) surface.

Bond dissociation	E_a (eV)	E_{rxn} (eV)
H-H	0.26	-1.22
Bond recombination	E_a (eV)	E_{rxn} (eV)
H-F	1.17	0.85
